# An individual nomogram can reliably predict tumor spread through air spaces in non-small-cell lung cancer

**DOI:** 10.1186/s12890-022-02002-1

**Published:** 2022-05-26

**Authors:** Shuai Wang, Huankai Shou, Haoyu Wen, Xingxing Wang, Haixing Wang, Chunlai Lu, Jie Gu, Fengkai Xu, Qiaoliang Zhu, Lin Wang, Di Ge

**Affiliations:** 1 Department of Thoracic Surgery, Zhongshan Hospital, Fudan University, Shanghai, 20032 China; 2 Department of Pathology, Zhongshan Hospital, Fudan University, Shanghai, 20032 China

**Keywords:** Lung cancer, Spread through air spaces, Predict, Nomogram

## Abstract

**Background:**

Tumor spread through air spaces (STAS) has been shown to adversely affect the prognosis of lung cancer. The correlation between clinicopathological and genetic features and STAS remains unclear.

**Method:**

We retrospectively reviewed 3075 NSCLC patients between2017-2019. We evaluated the relationship between STAS and patients’ clinicopathological and molecular features. The chi-square test was performed to compare categorical variables. Univariate analysis and multivariate logistic regression analysis were performed to investigate the association of clinical factors with STAS. A nomogram was formulated to predict the presence of STAS.

**Results:**

STAS was identified in 617 of 3075 patients (20.07%). STAS was significantly related to sex (*p* < 0.001), smoking (*p* < 0.001), CEA (*p* < 0.001), differentiation (*p* < 0.001), histopathological type (*p* < 0.001), lymphatic vessel invasion (*p* < 0.001), pleural invasion (*p* < 0.001), T stage (*p* < 0.001), N stage (*p* < 0.001), M stage (*p* < 0.001), and TNM stage (*p* < 0.001). STAS was frequently found in tumors with wild-type *EGFR* (*p* < 0.001), *KRAS* mutations (*p* < 0.001), *ALK* rearrangements (*p* < 0.001) or *ROS1* rearrangements (*p* < 0.001). For programmed death-1 (PD-1)/programmed death ligand-1 (PD-L1), STAS was associated with PD-L1 expression level in tumor cells (*p* < 0.001) or stromal cells (*p* < 0.001), while PD-1 only in stromal cells (*p* < 0.001). Multivariable analyses demonstrated significant correlations between STAS and CEA level (*p* < 0.001), pathological grade (*p* < 0.001), lymphatic vessel invasion (*p* < 0.001), pleural invasion (*p* = 0.001), and TNM stage (*p* = 0.002). A nomogram was formulated based on the results of the multivariable analysis.

**Conclusions:**

Tumor STAS was associated with several invasive clinicopathological features. A nomogram was established to predict the presence of STAS in patients with NSCLC.

**Supplementary Information:**

The online version contains supplementary material available at 10.1186/s12890-022-02002-1.

## Background

Currently, lung cancer has the highest mortality among malignant neoplasms in the world, accounting for approximately 1.8 million (18%) cancer-related deaths worldwide in 2020 [1]. Spread through air spaces (STAS) is considered to be a new invasion pattern of lung cancer in addition to blood and lymphatic vessel invasion, pleural invasion and direct invasion [2]. STAS consists of micropapillary clusters, solid nests, or single cells beyond the edge of the tumor into air spaces in the surrounding lung parenchyma. [3]

Recent studies have shown that STAS is associated with clinicopathologic features and suggests a poor clinical prognosis [4–8]. However, the relationship between STAS and genetic mutations remains unclear. The relationship between STAS and immune checkpoints [programmed death-1 (PD-1)/programmed death ligand-1 (PD-L1)] is still unknown. Therefore, further study is needed to clarify the correlation between molecular features and STAS.

Recently, STAS has been reported to be associated with poor prognosis in lobectomy as well as limited resection [10]. Besides, in early-stage adenocarcinoma with STAS, lobectomy was associated with better outcomes than sublobar resection [9]. Hence, it is important to identify STAS preoperatively or intraoperatively to help stratify patients for limited resection rather than lobectomy. However, it is still difficult for pathologists to accurately identify STAS on frozen sections intraoperatively*.* Walts et al*.* [11] reported that the sensitivity for STAS detecting was 50%, and the negative predictive value was only 8% on frozen sections. Therefore, we established a nomogram to predict STAS preoperatively based on patients’ clinical and intraoperative pathological features.

## Methods

### Patients

We reviewed patients with lung cancer in the Department of Thoracic Surgery of Zhongshan Hospital from October 2017 to August 2019. A total of 3397 consecutive patients who underwent surgical resection were studied. The patients enrolled had to meet the following inclusion criteria: (1) Pathological confirmation of primary NSCLC. (2) Sublobectomy, lobectomy or pneumonectomy with lymph node dissection was performed to achieve complete resection. (3) Negative surgical margins. The exclusion criteria were as follows: (1) Patients who underwent a needle biopsy of the tumor site before surgery. (2) Patients who received preoperative neoadjuvant therapy. (3) Patients with a history of previous lung surgery or other malignancies. According to these criteria, we identified a total of 3075 NSCLC cases. The pathologic stage was reclassified according to the 8th edition of the *American Joint Committee on Cancer Staging Manual*.

### Pathologic examination

**Fig. 1:**
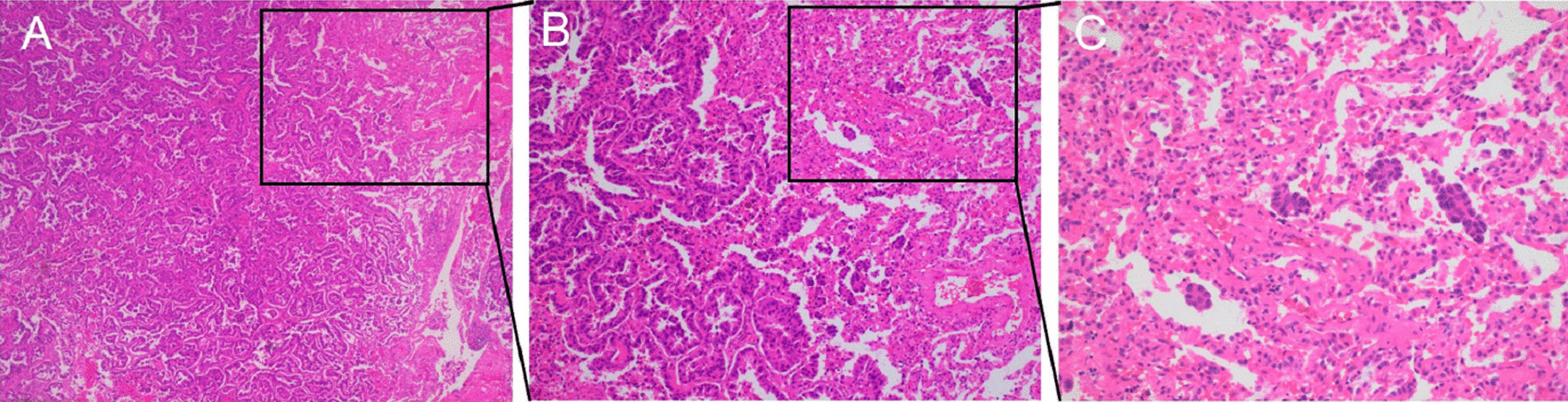
Representative images of tumor STAS. STAS was identified within the air spaces in the lung parenchyma, beyond the edge of the main tumor **A** HE × 50, **B** HE × 100, **C** HE × 200

All hematoxylin eosin slides were reviewed by at least two experienced pathologists who were blinded to patients’ clinical outcomes. Tumor STAS was defined as tumor cells either in clusters, solid nests or aggregates of single cells beyond the edge of the main tumor into airspaces in the surrounding lung parenchyma and separation from the main tumor [12]. A representative image of STAS is shown in Fig. 1.

### Immunohistochemistry (IHC)

IHC was carried out on formalin-fixed, paraffin-embedded tissue blocks according to the manufacturers’ instructions. IHC antibodies included those targeting p63 (clone 4A4, 1:200; Biocare), thyroid transcription factor-1 (TTF1) (clone 8G7G3/1, 1:50; Abcam), CK7 (EPR17078, 1:1000; Abcam), Napsin A (clone IP64, 1:200; Leica), PD-1 (NAT105; 1:100; ab52587; Abcam), PD-L1 (clone 28–8; 1:100; ab205921; Abcam) and PD-L1 (SP142; 1:100; ab228462; Abcam). IHC was performed as previously described [13]. The results were evaluated by two experienced pathologists who were blind to the patients’ clinical features. For p63, TTF1, CK7, and Napsin A, a positive score was based on moderate to strong staining observed by experienced pathologists.

For PD-1 and PD-L1, the expression was assessed in both tumor and stromal cells. Positive PD-1/PD-L1 expression was defined as staining of the cell membrane. PD-1/PD-L1 was validated quantitatively at specified levels of < 1%, 1–5%, 5–10% or ≥ 10% of tumor or stromal cells in a section that included at least 100 tumor or stromal cells. Since the expression of PD-1 was extremely low in tumor cells, the expression of PD-1 in tumors was grouped into two categories: < 1% and ≥ 1%.

### Gene mutation analysis

Genomic DNA or RNA was extracted according to the manufacturers’ instructions (RNeasy Mini Kit and QiAamp DNA Mini Kit, Qiagen, Hilden, Germany). The Revert Aid First Strand cDNA Synthesis Kit (Fermentas, St Leon-Rot, Germany) was used to reverse transcribe RNA samples into single-stranded cDNA. The mutational status of *EGFR* (exons 18–21), *HER2* (exons 18–21), *KRAS* (exons 2–3) and *BRAF* (exons 11–15) were identified using polymerase chain reaction (PCR)-based direct sequencing of cDNA and verified by DNA sequencing analysis. Mutation of *PIK3CA* (exons 9 and 20) was detected by sequencing of genomic DNA. A combination of reverse transcriptase PCR (RT-PCR) and quantitative real-time PCR (qRT-PCR) was used to detect *ALK*, *ROS1*, and *RET* fusions. All the fusions were further validated using fluorescent in situ hybridization (FISH).

### Statistical analysis

Comparisons of continuous variables were performed by one-way analysis of variance. Associations between categorical variables were investigated by the chi-square test. Univariate analysis and multivariate logistic regression analysis were performed to investigate the association of clinical factors with STAS. Only variables with *p* value < 0.05 in univariate analysis were entered in multivariate logistic regression analysis using forced entry. A nomogram was built based on the results of multivariate analysis. The prediction error was estimated with 100 bootstrap samples. The concordance index (C-index) and calibration plots were used to evaluate model performance. Nomogram plot calibration was used to estimate the overall agreement between the predicted and observed incidence of STAS. *P* value < 0.05 was considered to indicate statistical significance.

## Results

### Patient demographics and clinicopathological characteristics

In total, 3075 patients met the inclusion criteria. The patient characteristics are summarized in Table 1. According to the Union for International Cancer Control (UICC) staging system for NSCLC, there were 56 patients with stage 0 disease (1.82%), 2083 patients with stage IA disease (67.74%), 406 patients with stage IB disease (13.20%), 233 patients with stage II disease (7.58%) and 297 patients with stage III-IV disease (9.66%). Histologically, 2783 patients had adenocarcinoma histology (90.50%), 227 patients had squamous cell carcinoma histology (7.38%), and 65 patients had large cell carcinoma histology (2.11%). STAS was found in the histopathological slides of 617 patients (20.07%).

**Table 1:** Association of STAS with clinicopathologic characteristics

Data		All patients (n = 3075)	STAS		*p*
			Present (n = 617)	Absent (n = 2458)	
		N (%)	n (%)	n (%)	
*Patient characteristics*					
Age	≥ 60	1591(51.74)	377(23.70)	1214(76.30)	< 0.001
	< 60	1484(48.26)	240(16.17)	1244(83.83)	
Sex	Female	1663(54.08)	262(15.75)	1401(84.25)	< 0.001
	Male	1412(45.92)	355(25.14)	1057(74.86)	
Smoking	Never	2486(80.85)	458(18.42)	2028(81.58)	< 0.001
	Former/current	589(19.15)	159(26.99)	430(73.01)	
Tumor site	Left	1191(38.73)	234(19.65)	957(80.35)	0.809
	Right	1853(60.26)	376(20.29)	1477(79.71)	
	Bilateral	31(1.01)	7(22.58)	24(77.42)	
Surgery form	Thoracotomy	145(4.72)	47(32.41)	98(67.59)	< 0.001
	Thoracoscopic surgery	2930(95.28)	570(19.45)	2360(80.55)	
Surgery types	Sublobectomy	1232(40.07)	101(8.20)	1131(91.80)	< 0.001
	Lobectomy or pneumonectomy	1843(59.93)	516(28.00)	1327(72.00)	
CEA	< 5	2056(86.31)	260(12.65)	1796(87.35)	< 0.001
	≥ 5	326(13.69)	131(40.18)	195(59.82)	
*Pathological characteristics*					
Postoperative pathology	Squamous cell carcinoma	227(7.38)	40(17.62)	187(82.38)	0.604
	Adenocarcinoma	2783(90.50)	563(20.23)	2220(79.77)	
	Large Cell carcinoma	65(2.11)	14(21.54)	51(78.46)	
Pathological grade	well	534(17.37)	0(0)	534(100)	< 0.001
	moderately	1699(55.25)	265(15.60)	1434(84.40)	
	poorly	842(27.38)	352(41.81)	490(58.19)	
ADC type	AIS + MIA + LEP	573(20.59)	0(0)	573(100)	< 0.001
	ACI + VIA + PAP		1961(70.46)	407(20.75)	1554(79.25)
	SOL + LCA + MIP		249(8.95)	156(62.65)	93(37.35)
SCC type	keratinizing	119(52.42)	19(15.97)	100(84.03)	0.601
	Nonkeratinizing	108(47.58)	21(19.44)	87(80.56)	
Lymphatic & blood	−	2799(91.02)	448(16.01)	2351(83.99)	< 0.001
vessel invasion	+	276(8.98)	169(61.23)	107(38.77)	
Pleural invasion	−	2565(83.41)	398(15.52)	2167(84.48)	< 0.001
	+	510(16.59)	219(42.94)	291(57.06)	
T	Tis	56(1.82)	0(0)	56(100)	< 0.001
	T1	2227(72.42)	311(13.96)	1916(86.04)	
	T2	660(21.46)	243(36.82)	417(63.18)	
	T3	60(1.95)	25(41.67)	35(58.33)	
	T4	72(2.34)	38(52.78)	34(47.22)	
N	N0	2677(87.06)	399(14.90)	2278(85.10)	< 0.001
	N1	170(5.53)	89(52.35)	81(47.65)	
	N2	228(7.41)	129(56.58)	99(43.42)	
M	M0	3043(98.96)	593(19.49)	2450(80.51)	< 0.001
	M1	32(1.04)	24(75.00)	8(25.00)	
TNM	0	56(1.82)	0(0)	56(100)	< 0.001
	IA	2083(67.74)	226(10.85)	1857(89.15)	
	IB	406(13.20)	123(30.30)	283(69.70)	
	II	233(7.58)	106(45.49)	127(54.51)	
	III-IV	297(9.66)	162(54.55)	135(45.45)	
CK7	−	173(6.06)	21(12.14)	152(87.86)	0.01
	+	2680(93.94)	541(20.19)	2139(79.81)	
NapsinA	−	403(13.33)	85(21.09)	318(78.91)	0.641
	+	2621(86.67)	526(20.07)	2095(79.93)	
p63	−	1456(55.66)	322(22.12)	1134(77.88)	0.019
	+	1160(44.34)	213(18.36)	947(81.64)	
TTF-1	−	290(9.53)	55(18.97)	235(81.03)	0.645
	+	2752(90.47)	557(20.24)	2195(79.76)	

ADC, adenocarcinoma; *SCC*, squamous cell carcinoma; *AIS*, Adenocarcinoma in situ; *MIA*, minimally invasive adenocarcinoma; *LEP*, lepidic predominant; *ACI*, acinar predominant; *VIA*, variants of invasive adenocarcinoma; *PAP*, papillary predominant; *SOL*, solid predominant; *LCA*, lung cribriform adenocarcinoma; *MIP*, micropapillary predominant; *TNM*, tumor node metastasis

### Association between clinicopathological characteristics and STAS

Among the clinical features, the presence of STAS was significantly associated with age ≥ 60 (*p* < 0.001), male sex (*p* < 0.001), smoking (*p* < 0.001), thoracotomy (*p* < 0.001), lobectomy or pneumonectomy (*p* < 0.001) and CEA ≥ 5 ng/ml (*p* < 0.001) (Table 1).

The presence of STAS was found to be significantly associated with high pathological grade (*p* < 0.001). In adenocarcinoma, STAS was more frequently observed in more invasive subtypes (*p* < 0.001). Moreover, we found significant positive correlations between STAS and the pathological features of lymphatic and blood vessel invasion (*p* < 0.001), pleural invasion (*p* < 0.001), high T stage (*p* < 0.001), high N stage (*p* < 0.001), high M stage (*p* < 0.001) and advanced TNM stage (*p* < 0.001) (Table 1).

### Association between PD-1/PD-L1 and STAS

We further analyzed the correlation of STAS with PD-1/PD-L1 expression (SP28-8 and SP142) and found significant relationships between STAS and PD-L1 expression level in tumor cells (*p* < 0.001) or stromal cells (*p* < 0.001), while PD-1 expression was correlated with STAS only in stromal cells (*p* < 0.001). The results suggested that patients with PD-1 expression over 10% in stromal cells had a significantly higher rate of STAS. For PD-L1, an expression level between 5 and 10% in tumor cells or stromal cells was associated with a higher incidence of STAS (Table 2). We further analyzed the relationship between PD-L1 and *EGFR* mutation and found that patients without *EGFR* mutation had a significantly higher rate of PD-L1 expression (Table 3).

**Table 2:** Association of STAS with PD-1/PD-L1 expression

Data		All patients (n = 3075) n (%)	STAS		*p*
			Present (n = 617)	Absent (n = 2458)	
			n (%)	n (%)	
PD1 tumor cell	< 1%	2868 (98.86)	573 (19.98)	2295 (80.02)	0.661
	≥ 1%	33 (1.14)	5 (15.15)	28 (84.85)	
PD1 stromal cell	< 1%	510 (17.61)	54 (10.59)	456 (89.41)	< 0.001
	1–5%	1206 (41.64)	218 (18.08)	988 (81.92)	
	5–10%	697 (24.07)	166 (23.82)	531 (76.18)	
	≥ 10%	483 (16.68)	140 (28.99)	343 (71.01)	
PD-L1(SP28-8) tumor cell	< 1%	1879 (85.76)	311 (16.55)	1568 (83.45)	< 0.001
	1–5%	86 (3.93)	31 (36.05)	55 (63.95)	
	5–10%	48 (2.19)	18 (37.50)	30 (62.50)	
	≥ 10%	178 (8.12)	65 (36.52)	113 (63.48)	
PD-L1(SP28-8) stromal cell	< 1%	718 (33.04)	108 (15.04)	610 (84.96)	< 0.001
	1–5%	353 (16.24)	94 (26.63)	259 (73.37)	
	5–10%	139 (6.40)	53 (38.13)	86 (61.87)	
	≥ 10%	963 (44.32)	164 (17.03)	799 (82.97)	
PD-L1(SP142) tumor cell	< 1%	1588 (78.34)	255 (16.06)	1333 (83.94)	< 0.001
	1–5%	102 (5.03)	32 (31.37)	70 (68.63)	
	5–10%	67 (3.31)	28 (41.79)	39 (58.21)	
	≥ 10%	270 (13.32)	106 (39.26)	164 (60.74)	
PD-L1(SP142) stromal cell	< 1%	423 (20.91)	49 (11.58)	374 (88.42)	< 0.001
	1–5%	365 (18.04)	88 (24.11)	277 (75.89)	
	5–0%	245 (12.11)	85 (34.69)	160 (65.31)	
	≥ 10%	990 (48.94)	196 (19.80)	794 (80.20)	

PD-1, Programmed death-1; PD-L1, programmed death-ligand 1

**Table 3:** Association of *EGFR* mutation with PD-1/PD-L1 expression

Data		All patients (n = 2853) n(%)	EGFR		*p*
			Mutation (n = 1575)	Wild (n = 1278)	
			n (%)	n (%)	
PD-L1(SP28-8) tumor cell	< 1%	1787 (85.71)	1067 (59.71)	720 (40.29)	< 0.001
	1–5%	81 (3.88)	30 (37.04)	51 (62.96)	
	5–10%	45 (2.16)	15 (33.33)	30 (66.67)	
	≥ 10%	172 (8.25)	56 (32.56)	116 (67.44)	
PD-L1(SP142) tumor cell	< 1%	1477 (78.27)	886 (59.99)	591 (40.01)	< 0.001
	1–5%	98 (5.19)	47 (47.96)	51 (52.04)	
	5–10%	62 (3.29)	23 (37.10)	39 (62.90)	
	≥ 10%	250 (13.25)	71 (28.40)	179 (71.60)	

### Association between genetic mutations and STAS

The relationships between the genetic characteristics of tumors and STAS are summarized in Table 4. STAS was frequently observed in tumors with *KRAS* mutations (*p* < 0.001), *ALK* rearrangements (*p* < 0.001) or *ROS1* rearrangements (*p* < 0.001). In contrast, STAS was frequently observed in tumors without *EGFR* mutations (*p* < 0.001). Furthermore, *BRAF* mutation (*p* = 0.069), *PIK3CA* mutation (*p* = 0.749), *HER2* mutation (*p* = 0.853) and *RET* rearrangement (*p* = 0.489) were not associated with the presence of STAS. Univariate analysis revealed that age (*p* < 0.001), sex (*p* < 0.001), smoking (*p* < 0.001), CEA level (*p* < 0.001), pathological grade (*p* < 0.001), lymphatic vessel invasion (*p* < 0.001), pleural invasion (*p* < 0.001), T stage (i < 0.001), N stage (*p* < 0.001), M stage (*p* < 0.001) and TNM stage (*p* < 0.001) were significant factors. To rule out confounding factors, multivariate analysis was performed and suggested that CEA level (*p* < 0.001), pathological grade (*p* < 0.001), TNM stage (p = 0.002), lymphatic vessel invasion (*p* < 0.001) and pleural invasion (*p* = 0.001) were all independent factors of STAS (Additional File 1: Table S1).

**Table 4:** Association of STAS with genetic mutations

Data		All patients (n = 3075) n (%)	STAS		*p*
			Present (n = 617)	Absent (n = 2458)	
			n (%)	n (%)	
*Gene mutation types*					
EGFR mutant	−	1278 (44.79)	320 (25.04)	958 (74.96)	< 0.001
	+	1575 (55.21)	284 (18.03)	1291 (81.97)	
KRAS mutant	−	2728 (96.33)	541 (19.83)	2187 (80.19)	< 0.001
	+	104 (3.67)	59 (56.73)	45 (43.27)	
BRAF mutant	−	2764 (99.57)	543 (19.65)	2221 (80.35)	0.069
	+	12 (0.43)	5 (41.67)	7 (58.33)	
PIK3CA mutant	−	2761 (99.46)	546 (19.78)	2215 (80.22)	0.749
	+	15 (0.54)	2 (13.33)	13 (86.67)	
HER2 mutant	−	2727 (98.31)	538 (19.73)	2189 (80.27)	0.853
	+	47 (1.69)	10 (21.28)	37 (78.72)	
ALK genetic isolation	−	2730 (96.36)	555 (20.33)	2175 (79.67)	< 0.001
	+	103 (3.64)	46 (44.66)	57 (55.34)	
ROS1 genetic isolation	−	2820 (99.44)	588 (20.85)	2232 (79.15)	< 0.001
	+	16 (0.56)	14 (87.50)	2 (12.50)	
RET genetic isolation	−	2747 (98.96)	540 (19.66)	2207 (80.34)	0.489
	+	29 (1.04)	7 (24.14)	22 (75.86)	

### Predictive nomogram

**Fig. 2:**
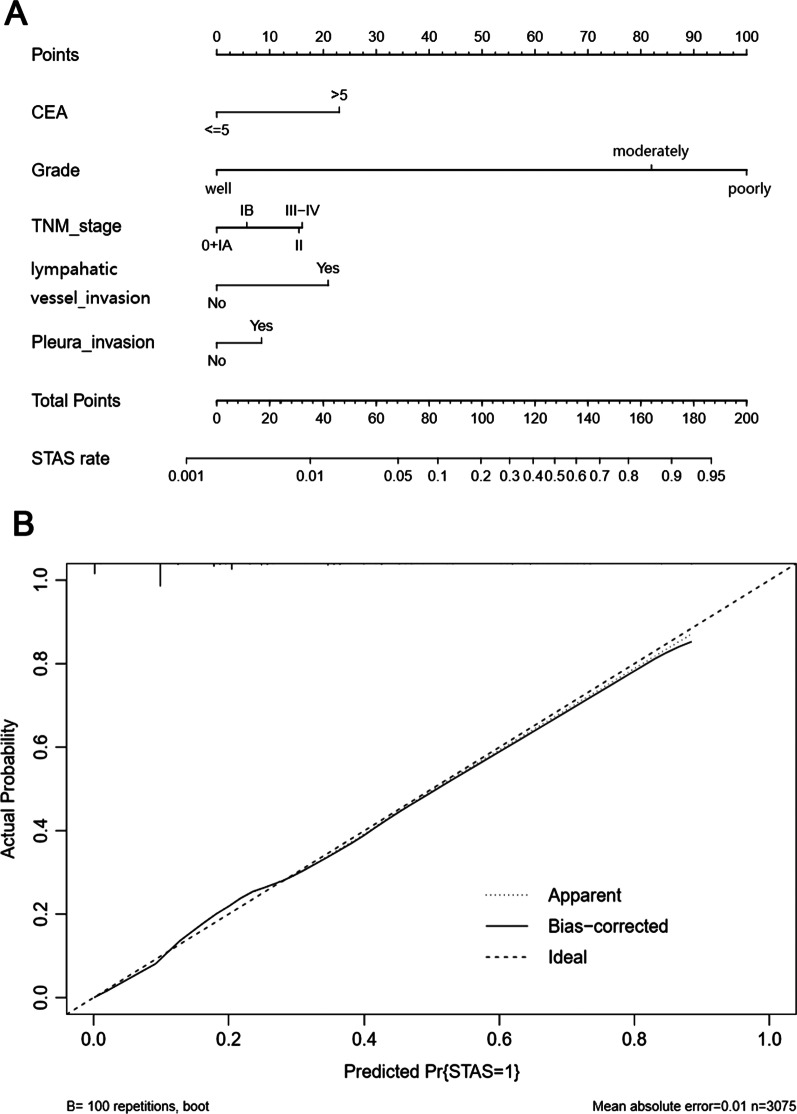
The nomogram for predicting the incidence of STAS in NSCLC patients. **A** Nomogram to predict the incidence of STAS. **B** Calibration curves of the nomogram predicting the incidence of STAS in NSCLC patients

The predictive nomogram, which was constructed based on the final multivariate model, is shown in Fig. 2A. The nomogram had a C-index of 0.826, which indicated good predictive ability. The calibration plot based on bootstrap resampling validation is showed in Fig. 2B and demonstrated good agreement of the actual and predicted STAS rates.

## Discussion

Our current study enrolled 3075 patients and demonstrated that STAS is associated with the clinicopathological and molecular features of resected NSCLC. In addition, this is the biggest study to find the correlation between STAS and PD-1/PD-L1. Notably, we constructed a nomogram to effectively and accurately predict the presence of STAS.

A large number of studies have described the incidence of STAS in NSCLC, but the positive rate of STAS ranged from 14.8% [14] to 60.5% [5]. The difference may result from the variable number of patients or the evaluation of STAS. In this retrospective study, we included 3075 patients with resected NSCLC, and the overall incidence of STAS was 20.07%.

It can be seen from the nomogram that the most important factor predicting STAS is pathological grade. Several studies have pointed out the relationship between STAS and pathological grade. In 2020, Zeng et al [15] matched 170 pairs of patients (340 cases) and found a significant difference between STAS and tumor differentiation but no difference in multivariate analysis. The results might be limited by the small sample size. In addition to the pathological grade, other predictive factors in the nomogram, such as CEA level, TNM stage, lymphatic vessel invasion and pleural invasion, have been reported to be associated with STAS in many studies [7, 16–18]. The nomogram visually simplifies the prediction of STAS and could help assess STAS on frozen sections.

The relationship between STAS and PD-1/PD-L1 expression remains unclear. To date, only one study has observed no significant difference between STAS and PD-L1 expression in resected pathological stage I lung adenocarcinoma [7]. In our research, we demonstrated the significant relationship between STAS and PD-1/PD-L1 expression for the first time. Recent study has demonstrated that a high density of CD68 + TAMs is an independent predictor of an increased STAS rate [19]. This finding suggested that STAS may be related to tumor immune environment. Since PD-1/PD-L1 are the key in the tumor immune environment, high PD-L1 might affect the occurrence of STAS through immune environment. But this conjecture needs further research to confirm. Assessing PD-L1 expression to predict the response to immune checkpoint inhibitor therapy is not straightforward. In different clinical trials of immune checkpoint inhibitor drugs, the evaluation of PD-L1 expression lacked uniformity, largely due to the unique anti-PD-L1 IHC assay [20]. The Blueprint PD-L1 Immunohistochemistry Project assessed 5 commercial PD-L1 IHC assays and found highly comparable staining with clone 22C3, clone 28–8 and clone SP263 assays; less sensitivity with clone SP142 assay; and higher sensitivity with clone 73–10 assay in terms of the detection of PD-L1 expression on tumor cells, while poor reliability was found on immune cells [21]. In the current study, we found that clone SP142 assay and clone SP28-8 assay showed different rate of STAS. These results suggested that different IHC assays assessing PD-L1 expression should be taken into consideration to help us make more accurate decisions.

In terms of genetic alterations, the relationship is largely unclear. According to previous studies, STAS was associated with *BRAF* mutations, *ALK* rearrangement and wild-type *HER2*. [17, 22–25] In the current study, 44.66% (46/103) of cases with *ALK* rearrangements demonstrated STAS. However, no significance was observed in the association of *BRAF* and *HER2* with STAS. One possible reason for this result might be the low rate of *BRAF* (12 cases) and *HER2* (47 cases) mutations in our research. For *KRAS* mutations, one study reported that STAS was frequently observed in tumors with *KRAS* mutations [17], while others found no significant association [15, 16, 22]. Our result revealed that STAS was correlated with *KRAS* mutations. Two studies observed an association between STAS and *ROS1* rearrangement. One identified that STAS was associated with *ROS1* rearrangement [26], while the other reported no relationships. [16] In our search, STAS was correlated with *ROS1* rearrangement. The association between STAS and *EGFR* mutations is complicated. In most reports, STAS was found to be more common in tumors with wild-type *EGFR*. [14, 16, 22, 23] Conversely, Szu-Yen Hu et al*.* pointed out that STAS was associated with *EGFR* mutations [17]. Moreover, no relationship was observed between STAS and *EGFR* in some studies [7, 15, 24]. In the current work, STAS was more frequently observed in tumors with wild-type *EGFR*. Besides, wild-type *EGFR* was associated with higher PD-L1 expression in our study and the result was in line with previous studies by others [27, 28]. However, some studies showed that PD-L1 expression was higher in patients with *EGFR* mutations [29, 30]. The relationship between PD-L1 and *EGFR* is conflict. The reason may be related to the heterogeneity of lung cancer. Further studies focused on tumor environment and driver oncogene is needed. Moreover, we also found that STAS was not associated with *PIK3CA* mutations or *RET* rearrangements, which have never been investigated before. However, as the rates of *PIK3CA* mutation and *RET* rearrangement are quite low, further research is needed to illustrate the relationship.

The mechanism of STAS remains unclear. STAS was found to be associated with high expression of metastasis-associated protein 1 (MTA1), which was reported to be associated with metastatic behaviors and poor prognosis [31]. Meng Jia et al [32] reported that STAS was significantly related to low E-cadherin and high vimentin expression in both lung adenocarcinomas and lung squamous cell carcinomas. Low E-cadherin expression and high vimentin expression are markers of epithelial-mesenchymal transition (EMT), which is often related to tumor invasion and metastasis [33, 34]. In the current research, STAS was significantly associated with lymphatic vessel invasion, pleural invasion and high TNM stage. These results demonstrated the aggressive biological features of STAS. STAS was also associated with PD-1/PD-L1 expression and several gene mutations in our study. Therefore, the mechanisms underlying STAS might be associated with EMT, the tumor environment and gene mutations. Further research is needed to clarify the mechanism of STAS.

As for surgical approach, it is generally thought that physical squeezing pressure during surgical procedure could affect the presence of STAS, especially pulling out the specimen through the tiny thoracoscopic port in thoracoscopic surgery. However, a prior prospective study by Blaauwgeers et al. [35] demonstrated no different incidences of loose fragments in different procedure groups (thoracotomy vs. thoracoscopic surgery). A recent study found higher incidence of STAS in lobectomy than limited resection [36]. Several other studies did not show significant relationship between STAS and surgical procedure (radical surgery vs. limited surgery) [12, 37]. In our research, STAS was more common in patients who underwent thoracotomy than thoracoscopic surgery, radical surgery than limited surgery. Since STAS is associated with higher stage NSCLC, the results might be due to the fact that the patients with higher stage NSCLC might undergo radical surgery and thoracotomy.

Whether STAS is a real in vivo phenomenon or an ex vivo artifact is disputable. During thoracoscopic surgery, resected lung specimens containing tumors are squeezed through tiny thoracoscopic port, which may cause tumor cells to detach at the tumor periphery and move to adjacent air spaces [2]. In the current research, STAS was more frequently observed when thoracotomy was performed. In addition, numerous studies have shown that STAS is independently associated with a poor prognosis in NSCLC patients [5–8]. These findings support that STAS is a real and significant biological phenomenon rather than fake artifacts.

The current study has several limitations. First, the study was limited by its retrospective nature, and the data were obtained from a single institution. Second, our study lacked prognostic data due to the short postoperative follow-up time. In addition, we investigated only the clinicopathological and molecular features of NSCLC. Additional meaningful factors that may influence the prediction of STAS, such as morphologic subtypes of STAS, quantitative evaluation of STAS and radiology of STAS, might be missing from this analysis.

## Conclusions

In conclusion, the current study indicated that STAS was closely related to many clinical features and pathological characteristics of NSCLC patients. In addition, we demonstrated the relationship between STAS and PD-1/PD-L1 expression. STAS was also found to be associated with wild-type *EGFR*, *KRAS* mutation, *ALK* rearrangement and *ROS1* rearrangement. Finally, we built a nomogram that could predict the presence of STAS during surgery.

## Supplementary Information

**Additional file 1.** **Table S1.** Multivariate analysis of factors associated with tumor STAS.

## Data Availability

The data used in the current study are available from the corresponding author on reasonable request.

## Abbreviations

STAS: Tumor spread through air spaces
NSCLC: Non-small cell lung cancer
PD-1: Programmed death-1
PD-L1: Programmed death ligand-1
IHC: Immunohistochemistry
ADC: Adenocarcinoma
SCC: Squamous cell carcinoma
AIS: Adenocarcinoma in situ
MIA: Minimally invasive adenocarcinoma
LEP: Lepidic predominant
ACI: Acinar predominant
VIA: Variants of invasive adenocarcinoma
PAP: Papillary predominant
SOL: Solid predominant
LCA: Lung cribriform adenocarcinoma
MIP: Micropapillary predominant
TNM: Tumor node metastasis
