# Supplementary material for: An individual nomogram can reliably predict tumor spread through air spaces in non-small-cell lung cancer
Source: BMC Pulm Med. 2022 May 26;22:209. doi: 10.1186/s12890-022-02002-1 (PMC9137206; doi:10.1186/s12890-022-02002-1)
Supplement: Supplementary file 1 — Additional file 1. Table S1. Multivariate analysis of factors associated with tumor STAS. [file 12890_2022_2002_MOESM1_ESM.docx]

|  | **Likelihood Ratio Tests** | |
| --- | --- | --- |
| **Effect** | **Chi-Square** | **Sig.** |
| Sex | 3.535 | 0.060 |
| Age | 0.109 | 0.741 |
| Smoking | 2.316 | 0.128 |
| **CEA** | 79.674 | **0.000** |
| **Pathological grade** | 172.994 | **0.000** |
| **TNM** | 15.252 | **0.002** |
| **Lymphatic vessel invasion** | 43.078 | **0.000** |
| **Pleural invasion** | 11.047 | **0.001** |

Additional File1: Table S1. Multivariate analysis of factors associated with tumor STAS.
